# Cyclin-dependent kinases

**DOI:** 10.1186/gb4184

**Published:** 2014-06-30

**Authors:** Marcos Malumbres

**Affiliations:** 1 Cell Division and Cancer Group, Spanish National Cancer Research Centre (CNIO), Melchor Fernández Almagro 3, Madrid E-28029, Spain

## Abstract

**Summary:**

Cyclin-dependent kinases (CDKs) are protein kinases characterized by needing a separate subunit - a cyclin - that provides domains essential for enzymatic activity. CDKs play important roles in the control of cell division and modulate transcription in response to several extra- and intracellular cues. The evolutionary expansion of the CDK family in mammals led to the division of CDKs into three cell-cycle-related subfamilies (Cdk1, Cdk4 and Cdk5) and five transcriptional subfamilies (Cdk7, Cdk8, Cdk9, Cdk11 and Cdk20). Unlike the prototypical Cdc28 kinase of budding yeast, most of these CDKs bind one or a few cyclins, consistent with functional specialization during evolution. This review summarizes how, although CDKs are traditionally separated into cell-cycle or transcriptional CDKs, these activities are frequently combined in many family members. Not surprisingly, deregulation of this family of proteins is a hallmark of several diseases, including cancer, and drug-targeted inhibition of specific members has generated very encouraging results in clinical trials.

## Gene organization and evolutionary history

Cyclin-dependent kinases (CDKs) are serine/threonine kinases whose activity depends on a regulatory subunit - a cyclin. Based on the sequence of the kinase domain, CDKs belong to the CMGC group of kinases (named for the initials of some members), along with mitogen-activated protein kinases (MAPKs), glycogen synthase kinase-3 beta (Gsk3β), members of the dual-specificity tyrosine-regulated kinase (DYRK) family and CDK-like kinases [1]. In related kinases such as MAPKs, substrate specificity is conferred by docking sites separated from the catalytic site, whereas CDKs are characterized by dependency on separate protein subunits that provide additional sequences required for enzymatic activity. To aid nomenclature and analysis of CDKs, proteins belonging to this family have been recently renamed as Cdk1 through to Cdk20 [2].

CDKs were first discovered by genetic and biochemical studies in model organisms such as yeasts and frogs (reviewed in [3]). This work established the importance of CDKs in promoting transitions through the cell cycle. In addition, these studies showed that the catalytic subunit, the CDK, must associate with a regulatory subunit, the cyclin, whose protein levels are subject to regulation during the cell cycle (this oscillation lent these regulators their cyclin name). Since these pioneer studies conducted in the 1980s, the importance of CDKs acting as a major eukaryotic protein kinase family involved in the integration of extracellular and intracellular signals to modulate gene transcription and cell division has been clearly established [3-6].

**Figure 1:**
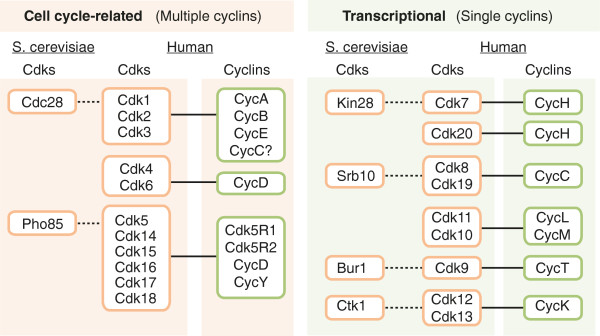
**Comparison of yeast and mammalian CDKs.** Cells of the budding yeast *Saccharomyces cerevisiae* contain two cell-cycle-related CDKs that are activated by multiple cyclins - Cdc28 and Pho85. Cdk1 is the mammalian ortholog of Cdc28, whereas Cdk5 is considered to be the Pho85 ortholog. The Cdk4/Cdk6 subfamily is not present in yeast. Kin28, Srb10, Bur1 and Ctk1 are the yeast orthologs of Cdk7, Cdk8, Cdk9 and Cdk12, respectively. The Cdk20 and Cdk11/Cdk10 subfamilies are not represented in yeast. Also indicated is the cyclin partner for the mammalian CDKs. CDK, cyclin-dependent kinase.

Despite their function in eukaryotic cell division and transcription, CDKs have undergone an extraordinary degree of evolutionary divergence and specialization. Six different CDKs are present in budding yeast (Figure 1). These CDKs can be grouped as, first, CDKs that bind multiple cyclins and can regulate the cell cycle and, second, CDKs that are activated by a single cyclin and are involved in the regulation of transcription. In the budding yeast *Saccharomyces cerevisiae*, the first group contains Cdc28 and Pho85, each binding nine or ten different cyclins, respectively. This promiscuity forms the basis for their dynamic regulation and their ability to phosphorylate multiple substrates, thus regulating the cell-division cycle in response to different cellular cues. The second group comprises four CDKs - Kin28, Srb10, Bur1 and Ctk1 - each activated by a single specific cyclin (Figure 1). These cyclins are usually not regulated in a cell-cycle-dependent manner, and the members of this second group of CDKs are involved in the control of gene transcription.

**Figure 2:**
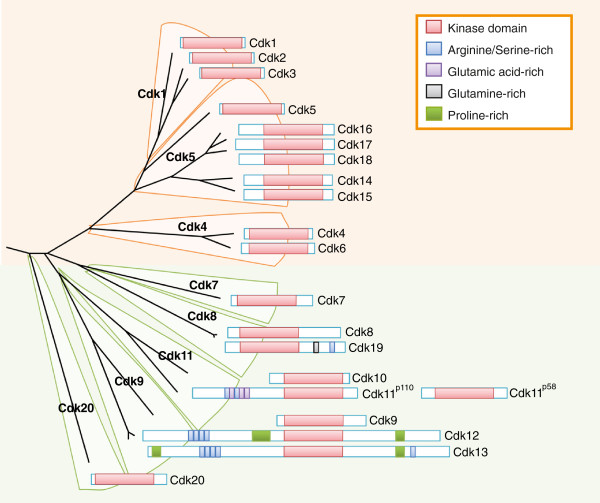
**Evolutionary relationships among the mammalian CDK subfamilies.** The name of the different CDK subfamilies functioning in the cell cycle (orange) or transcription (green) is shown in boldface, and the domain structure of the individual proteins is depicted. The conserved protein kinase domain (red) and some additional domains (see key) are indicated for each CDK. Human cells contain two separate genes, *Cdk11A* and *Cdk11B*, each of them encoding a long isoform, Cdk11^p110^, and a shorter protein, Cdk11^p58^, generated by an internal ribosome binding site. The phylogenetic tree is based on the comparison of the human kinase domains [1]. CDK, cyclin-dependent kinase.

The number of CDKs increased during evolution and was marked by a greater expansion of the cell-cycle-related group. Fungi contain 6 to 8 CDKs and 9 to 15 cyclins, whereas flies and echinodermata contain 11 CDKs and 14 cyclins, and human cells have 20 CDKs and 29 cyclins (Box 1) [7]. Evolutionary studies suggest that CDKs fall into eight subfamilies represented by Cdk1, Cdk4 and Cdk5 (from the yeast cell-cycle-related CDKs), and Cdk7, Cdk8, Cdk9, Cdk11 and Cdk20 (functioning as transcriptional CDKs) [7,8] (Figure 2). Like its yeast ortholog, Cdk1 is the only CDK essential for the cell cycle in mammals [9], whereas both Cdk2 and Cdk3 are dispensable [3,10]. Although Pho85 is not essential in yeast, this kinase is required for viability in some stress conditions, such as growth after starvation. Pho85 displays multiple cell-cycle-related functions as well as regulation of gene expression, metabolism, morphogenesis, cell polarity and aging; it functions as an integrator of signals such as nutrient availability, DNA damage or other types of stress [11]. Sequencing and functional studies suggest that the mammalian homolog of Pho85 is Cdk5, although these kinases cluster with multiple mammalian kinases of the Cdk5 subfamily, namely Cdk14 to Cdk18. Pho85 can interact with up to 10 cyclins of the Pcl1/Pcl2 or Pho80 groups, whereas mammalian Cdk5 is activated by non-cyclin proteins, including Cdk5R1 (p35) and Cdk5R2 (p39). Interestingly, other members of the Cdk5 subfamily, such as Cdk14 or Cdk16, are activated by cyclin Y, which is a cyclin closely related to yeast Pcl1/Pcl2 proteins [12,13]. The Cdk4 subfamily is unique as it is only found in eumetazoans, and the members of this family diverge equally from the Cdk1 or Cdk5 subfamilies (Figures 1 and 2) [7]. Other cell-cycle-related subfamilies, such as the Cdk1-related B-type CDKs, are plant specific and are not found in animals or fungi [14].

Transcriptional CDKs are more conserved, both in sequence and function (Figure 1). Yeast Kin28 and human Cdk7 are subunits of transcription factor TFIIH, which is involved in transcription initiation by phosphorylating the Ser5 residue of the RNA polymerase II (RNAPII) C-terminal domain (CTD) at gene promoters. Cdk7 is also able to phosphorylate and activate other CDKs, thus acting as a CDK-activating kinase (CAK; Box 2). Kin28 does not have this activity, which is mediated in yeast by a different kinase unrelated to CDKs, Cak1 [8]. The yeast protein Srb10 is orthologous to human Cdk8 and Cdk19 and is the enzymatic component of the Mediator complex involved in the regulation of RNAPII during transcription [15]. Cdk9 is the yeast Bur1 ortholog, whereas the function of yeast Ctk1 in the phosphorylation of the RNAPII CTD is performed by Cdk12 in *Drosophila* and in human cells [16]. The evolutionary relationship of the Cdk11 and Cdk20 subfamilies to the yeast CDKs is not clear, although these proteins are well conserved [7]. Unlike cyclins for cell-cycle-related kinases, the cyclin subunits of transcriptional CDKs do not show significant oscillations in protein levels during the cell cycle, and these transcriptional CDKs are therefore regulated by protein-protein interactions or other mechanisms. Transcription-related kinases possibly originated after cell-cycle-related CDKs and became more diverse as the complexity of transcription increased [17].

## Characteristic structural features

Like other CMGC kinases, CDKs are proline-directed serine/threonine-protein kinases with some preference for the **S/T**-P-X-K/R sequence as a consequence of the presence of a hydrophobic pocket near the catalytic site that accommodates the proline (position +1). However, the requirement for the basic residue in the +3 position is not maintained in Cdk4 or transcriptional CDKs, which display a less-stringent **S/T**-P-X consensus. Some other family members such as Cdk7 or Cdk9 are not necessarily proline directed and can also phosphorylate residues in the absence of the +1 proline [18].

**Figure 3:**
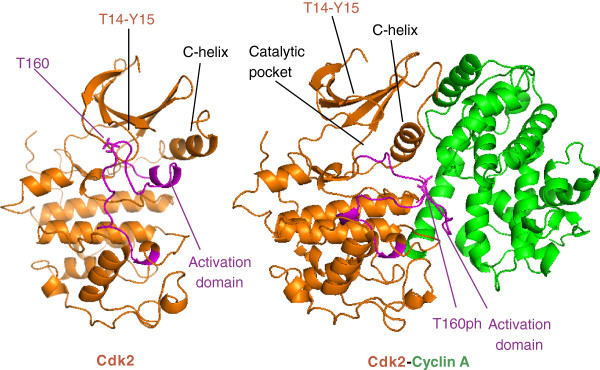
**A three-dimensional view of CDK structure and activation.** In monomeric Cdk2 (left; [PDB:1HCL]), the major C-helix (N-lobe) and the activation domain are close, ensuring that the catalytic pocket is inaccessible. Upon binding of cyclin A (right: [PDB: 1JST]), the C-helix and the activation domain are pulled apart - a configuration that is further fixed by phosphorylation of residue T160, making the catalytic pocket accessible for enzymatic activity. The position of the inhibitory Thr14 (T14) and Tyr15 (Y15) residues in the G-loop is also shown. Color code: CDK subunit, orange; cyclin subunit, green; purple indicates specific named protein domains. CDK, cyclin-dependent kinase.

The CDKs range in size from approximately 250 amino acid residues, just encompassing the catalytic serine/threonine kinase domain, to proteins of more than 1,500 residues, with amino- and/or carboxy-terminal extensions of variable lengths (Figure 2). Like all kinases, CDKs have a two-lobed structure. The amino-terminal lobe contains beta-sheets, whereas the carboxy-terminal lobe is rich in α-helices, and the active site is sandwiched in-between. The N-lobe contains a glycine-rich inhibitory element (G-loop) and a unique major helix - the C-helix (containing the PSTAIRE sequence in Cdk1). The C-lobe contains the activation segment, which spans from the DFG motif (D145 in Cdk2; EMBL:AK291941) to the APE motif (E172 in Cdk2) and includes the phosphorylation-sensitive (T160 in Cdk2) residue in the so-called T-loop (Figure 3). In the cyclin-free monomeric form the CDK catalytic cleft is closed by the T-loop, preventing enzymatic activity. In addition, the activation segment in the C-lobe - a platform for binding of the phospho-acceptor Ser/Thr region of substrates - is partially disordered.

## Cyclin-dependent kinase activation

Upon binding of the cyclin to Cdk2, the CDK C-helix packs against one specific helix in the cyclin partner through a surface characterized by extensive hydrophobic interactions. Association of cyclins to the C-helix promotes a rotation in the axis of this segment, generating new interactions that are part of the active ATP-binding site. In addition, cyclins take the C-lobe activation segment out of the catalytic site so that the threonine becomes accessible for activating phosphorylation by CAK (Figure 3). This phospho-threonine acts as a rigidifying hub, stabilizing the activated form of the kinase heterodimer [18,19]. The extent of the CDK-cyclin interface varies in the structure of Cdk4, Cdk9 or yeast Pho85 [18,20,21]. For instance, Cdk2 and cyclin A contact each other at both the N- and C-lobes, whereas the contacts between Cdk4 and cyclin D are limited to the N-lobe, and, unlike Cdk2, the cyclin does not impose an active conformation on the kinase as the Cdk4 ATP-binding site is still inaccessible to its substrates, even in the presence of the cyclin [20,21]. How Cdk4 becomes active is not completely clear, although the binding of the substrate is thought to induce the activation segment to open and to fit to the phospho-acceptor site. Some CDKs, such as Cdk5 or its yeast ortholog Pho85, do not require phosphorylation in the activation segment for activity, and these kinase can adopt the correct conformation through other interactions [18].

In addition to the consensus kinase domain, a few CDKs contain additional domains with functional relevance. Cdk16, Cdk17 and Cdk18 (containing a PCTAIRE sequence in the C-helix) are characterized by a conserved catalytic domain flanked by amino- and carboxy-terminal extensions involved in cyclin binding. Phosphorylation of the Cdk16 amino-terminal domain blocks binding to cyclin Y, providing a novel mechanism for regulation of these complexes [22]. In Cdk12 and Cdk13 (characterized by a PITAIRE motif), the kinase domain is localized in the center, and additional Arg/Ser-rich motifs in the amino terminus serve as docking sites for the assembly of splicing factors and regulators of splicing (Figure 2). These two kinases also contain proline-rich motifs, mostly concentrated in their carboxy-terminal region, that serve as binding sites for Src-homology 3 (SH3), WW or profilin-domain-containing proteins [16].

## Cyclin-dependent kinase inhibition

The glycine-rich region (G-loop) in the N-lobe is an additional regulatory region as it contains residues (Thr14 and Tyr15 in Cdk2; Figure 3) whose phosphorylation inhibits kinase activity. Phosphorylation of Thr14 and/or Tyr15 residues by Wee1 and Myt1 kinases inhibits several family members, preventing cell-cycle progression, for instance, in response to DNA damage. Elimination of these phosphates by phosphatases of the Cdc25 family is then required for activation of CDKs and cell-cycle progression [3,23]. Inhibitory phosphorylation at Thr14 and Tyr15 does not result in major changes in the CDK structure, but does inhibit the CDK activity by reducing the affinity of the CDK for its substrates. However, phosphorylation at Tyr15 seems to be activating in the case of Cdk5, perhaps by improving substrate recognition [18]. These residues are not present in Cdk7, in agreement with the general belief that this kinase is constitutively active and regulated at different levels.

Cell-cycle-related CDKs can also be negatively regulated by binding to small proteins of the INK4 or Cip/Kip families of inhibitors [19,24]. INK4 proteins (p16^*INK4a*^, p15^*INK4b*^, p18^*INK4c*^ and p19^*INK4d*^) are specific for the Cdk4 subfamily and interact with the monomeric CDKs. They function by distorting the cyclin interface and the ATP-binding pocket, thus preventing activation of Cdk4 and Cdk6 by D-type cyclins or by CAK [24]. Members of the Cip/Kip family of inhibitors (p21^*Cip1*^, p27^*Kip1*^ and p57^*Kip2*^) contact both the CDK and cyclin subunits and are able to inhibit CDK-cyclin heterodimers, giving additional levels of regulation once these complexes have already formed [19].

## Localization and function

### Cdk1 and Cdk4 subfamilies

**Figure 4:**
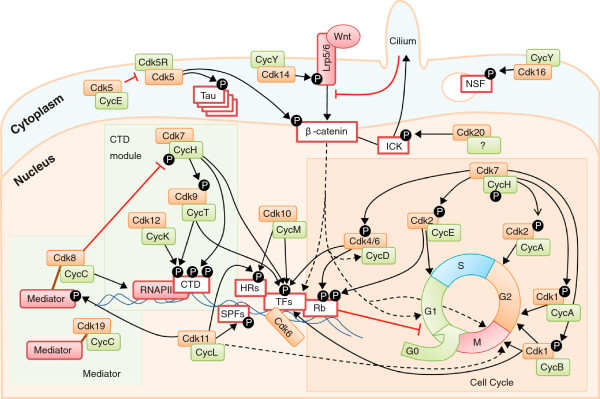
**An overview of CDK functions in the cell.** Each CDK (in orange boxes) is shown in a complex with its major partner (green) - for clarity, only a few substrates are depicted. Most CDKs function in the nucleus (orange background), whereas a few family members are attached to the cell membrane or display cytoplasmic activities (blue background). Classical cell cycle CDKs - Cdk4, Cdk6, Cdk2 and Cdk1 - regulate the transitions through the different phases of the cell-division cycle. These activities are at least partially mediated by the control of multiple transcription factors (TFs) or regulatory elements such as the retinoblastoma protein (Rb). Cdk10 and Cdk11 also control transcription by phosphorylating TFs, hormone receptors and associated regulators (HRs), or splicing factors (SPFs). Cdk7, Cdk9 and Cdk12 directly phosphorylate the C-terminal domain (CTD) of RNA polymerase II (RNAPII), thus modulating the different phases of generation of transcripts. The Mediator complex is specifically regulated by Cdk8 or the highly related Cdk19. Cdk7 functions as a CDK-activating kinase (CAK) by directly phosphorylating several of the CDKs mentioned above. Cdk5 displays many functions in the cell, but it is better known for its function in the control of neuron-specific proteins such as Tau. The members of the Cdk14 subfamily, such as Cdk14 itself or Cdk16, are activated at the membrane by cyclin Y and also participate in many different pathways, such as Wnt-dependent signaling or signal transduction in the primary cilium. It is important to note that, for clarity, many interactions between CDKs and other partners, substrates or cellular processes are not shown - for instance, Cdk1 can bind to other cyclins and can also phosphorylate more than 100 substrates during mitotic entry that are not indicated here. CAK, CDK-activating kinase; CDK, cyclin-dependent kinase; CTD, C-terminal domain; Rb, retinoblastoma protein; RNAPII, RNA polymerase II; SPF, splicing factor; TF, transcription factor.

The general picture in mammalian cells is that Cdk4 and Cdk6, upon transcriptional induction of D-type cyclins in response to several mitogenic stimuli, promote entry into the cell cycle (Figure 4) [25]. These kinases phosphorylate and inactivate the retinoblastoma protein (Rb), an adaptor protein that assembles different protein and protein-DNA complexes that repress transcription in response to a wide range of control mechanisms [25]. In human cells, Rb contains 13 conserved sites that are phosphorylated by CDKs in proliferating cells. Complexes between cyclin D and Cdk4 or Cdk6 phosphorylate residues Ser807 and Ser811, priming Rb for further phosphorylation by these or other CDKs at other sites [26]. CDK-dependent inactivation of Rb (or its relatives p107 and p130) results in de-repression of multiple genes encoding proteins required for DNA synthesis (S phase) or mitosis [25]. The activity of Cdk2 might also contribute to this process, although this kinase could have additional functions in DNA replication or DNA repair. Once cells have duplicated their DNA, Cdk1 becomes activated by A- and B-type cyclins, promoting cellular processes such as centrosome maturation and separation, chromosome condensation and mitotic entry after nuclear envelope breakdown [3]. This simplified view is obscured owing to multiple non-consensus interactions between CDKs and cyclins and compensatory roles [6]. For instance, when Cdk4 and Cdk6 are absent, Cdk2 can bind to D-type cyclins [27]. Cdk1 can also bind to cyclin E or cyclin D in the absence of Cdk2 or Cdk4, respectively [9], suggesting a scenario reminiscent of the yeast cell cycle in which Cdc28 is sufficient to induce all cell-cycle transitions by interacting with different cyclins [6].

### Cdk5 subfamily and cyclin-Y-related kinases

Despite its similarity to other cell-cycle-related Cdks, Cdk5 is the prototype of what are termed atypical CDKs. This kinase is activated by the non-cyclin proteins Cdk5R1 (p35) or Cdk5R2 (p39), and phosphorylation in the T-loop is not required for its activation [28,29]. Although Cdk5 is expressed in multiple cell types, its activity is thought to be more restricted owing to the expression of its activators p35 and p39 in terminally differentiated cells such as neurons [28]. However, in addition to its crucial functions in neuronal biology, Cdk5 plays multiple roles in gene expression, differentiation, angiogenesis and senescence, among others [5,28,29].

Interestingly, the Cdk5 activators carry an amino-terminal myristoylation motif that is required for their membrane targeting (Figure 4). Until recently, Cdk5 was thought to be the only membrane-associated Cdk, but recent data suggest that the CDKs Cdk14 to Cdk18 (PFTAIRE and PCTAIRE kinases) display similar activities upon binding to cyclin Y. Like Cdk5, Cdk16 requires no T-loop phosphorylation, suggesting that cyclin Y, like p35, tightly interacts with the activation loop, alleviating the need for an activating phosphorylation [13]. Cyclin Y is also N-myristoylated, and cyclin-Y-dependent recruitment and activation of Cdk14 at the plasma membrane results in phosphorylation of the Wnt co-receptor Lrp5/Lrp6 (Figure 4). Cdk16 also binds to cyclin Y, and these complexes phosphorylate several proteins, including N-ethylmaleimide-sensitive factor (NSF) for the control of exocytosis [30], and are essential for spermatogenesis [22]. The partner CDKs of cyclin Y display overlapping roles as knockdown of individual CDKs in *Xenopus* embryos failed to produce a phenotype, whereas depletion of cyclin Y and its highly related homolog cyclin-Y-like resulted in a Wnt loss-of-function phenotype [31]. In addition to the relevance of the Wnt pathway in the control of transcription, β-catenin and other Wnt regulators localize to centrosomes and/or kinetochores and regulate the formation and orientation of the mitotic spindle and the process of chromosome segregation [31]. In fact, cyclin Y reaches maximum levels at G2-M phase of the cell cycle and is degraded in a ubiquitin-dependent manner, similarly to mitotic cyclins, suggesting a crucial role for the cyclin-Y-Wnt pathway during cell division [12]. It is interesting to note that CDKs and cyclins of this subfamily, such as Cdk17 or cyclin Y, are highly conserved, at levels similar to Cdk1 or cyclin B [13]. In most cases, the cellular relevance of many Cdk5-subfamily members remains to be established.

### Control of RNA polymerase II by transcriptional cyclin-dependent kinases

One of the most important activities of CDKs is reversible phosphorylation of the CTD of the largest subunit (Rpb1) of RNAPII (Figure 4). The CTD consists of multiple repeats of an evolutionarily conserved heptapeptide possessing the consensus sequence Tyr-Ser-Pro-Thr-Ser-Pro-Ser, with the number of repeats varying among different organisms, ranging from 26 repeats in yeast to 52 in mammals. The CTD is the target of multiple posttranslational modifications, including phosphorylation, generating a complex regulatory code known as the CTD code. The CTD regulates the cycling of RNAPII between a hypophosphorylated form, able to enter the preinitiation complex, and a hyperphosphorylated form capable of processive elongation of the transcript [32]. Multiple CDKs can phosphorylate the CTD, including cell-cycle-related kinases Cdk1 or Cdk2 and most transcriptional CDKs of the Cdk7, Cdk8 and Cdk9 subfamilies (Figure 4). Cdk7 is a member of the ten-subunit general transcription factor TFIIH^b^ that phosphorylates Ser5 and Ser7 of the heptad during initiation and promoter clearance [33,34]. Cdk7 also phosphorylates and activates Cdk9, thus promoting downstream events [34]. To release the paused RNAPII and allow productive elongation, Ser2 of the heptad is then phosphorylated, a process in which both Cdk9 and Cdk12 have been implicated. Cdk9 binds to T-type cyclins (T1 and T2) as a subunit of the positive transcription elongation factor b (P-TEFb) that stimulates elongation. Cdk9 is the ortholog of Bur1, which contributes to phosphorylation of the Ser2 mark at the 5′ ends of genes [16,35]. Although Cdk9 was thought to be the major Ser2 kinase required for efficient elongation, recent data suggest that this requirement is mediated by a second substrate of Cdk9, the elongation factor subunit Spt5, whose Cdk9-dependent phosphorylation relieves the early pausing step [35]. Recent studies in *Drosophila* and human cells suggest that Cdk12, in complex with cyclin K, is the yeast Ctk1 ortholog responsible for most of the Ser2 phosphorylation at the CTD and especially the phosphorylation at promoter-distal regions [36,37]. Depletion of Cdk12 resulted in defective Ser2 phosphorylation at a subset of genes - mostly long and complex ones - but not a change in the rate of global transcription. Cdk12 is specifically required for the transcription of genes involved in the response to DNA damage, establishing a new link between the transcriptional machinery and cell-cycle regulation [37]. Cdk1 can also phosphorylate the CTD, and this activity is thought to inhibit transcription, although its physiological relevance has not been established. Transcript termination results in dephosphorylation of RNAPII, making it ready for another round of re-initiation. Although the control of dephosphorylation is not well understood, several CDK-counteracting phosphatases such as Cdc14 are likely to be involved [38,39].

Cdk8 and its closely related family member Cdk19 associate with C-type cyclins as part of the multi-subunit Mediator complex (Figure 4) [15]. This complex functions as a bridge linking gene-specific activators to the general RNAPII transcription machinery at the promoter, thus influencing nearly all stages of transcription and coordinating these events with changes in chromatin organization. Cdk8 (or Cdk19), along with cyclin C, Med12 and Med13, form the so-called Cdk8 module characteristic of the free Mediator form, devoid of RNAPII. The Cdk8 module responds to several intracellular signaling pathways, and it is commonly associated with repression of transcription, although it can also activate transcription [15]. Cdk8 has multiple targets and phosphorylates several transcription factors, affecting their stability and activity. Recent evidence suggests various roles in gene activation in the p53 network, the Wnt-β-catenin pathway, the serum-response network and other pathways governed by Smads or the thyroid hormone receptor [40]. Cdk8 also modulates Cdk7 activity by phosphorylating cyclin H, thus impeding Cdk7 activity and inhibiting initiation of transcription [33]. Finally, Cdk19 associates with similar Mediator complexes, although these complexes are likely to possess a specificity that is yet to be established [41].

### Cdk11 and Cdk20 subfamilies

Cdk11 proteins are the products of two highly related genes in mammals (*CDK11A* and *CDK11B*) encoding Cdk11A^p110^ and Cdk11B^p110^[2], as well as two smaller alternative proteins, Cdk11A^p58^ and Cdk11B^p58^, resulting from translation from an internal ribosome-binding site generated during G2-M phase. Cdk11 binds to L-type cyclins and participates in the coordination between transcription and RNA processing, particularly alternative splicing [42]. In budding yeast, Cdk11 has been shown to be a crucial factor for the interaction of the Cdk8 module with the Mediator complex through phosphorylation of conserved residues of the Med27 and Med4 Mediator subunits (Figure 4) [43]. Cdk11 also participates in many other pathways, such as hormone receptor signaling or autophagy [44-46]. The short isoform of Cdk11, Cdk11^p58^, is specifically expressed at G2-M, and its kinase activity is required for duplication of the centrioles, spindle dynamics and sister chromatid cohesion at centromeres during mitosis [47-49]. Lack of Cdk11 results in mitotic defects in mouse embryos, highlighting the crucial role of this ‘transcriptional’ kinase in the cell cycle [3].

Cdk10 is activated by cyclin M, a cyclin mutated in STAR syndrome, a developmental abnormality characterized by toe syndactyly, telecanthus and anogenital and renal malformations [50]. Cdk10-cyclin-M phosphorylates Ets2, promoting its degradation by the proteasome [50]. STAR-associated mutations in the gene encoding cyclin M impair binding of cyclin M to Cdk10, resulting in increased Ets2-dependent transcription of c-Raf and over-activation of the MAPK pathway. In the insect *Helicoverpa armigera*, Cdk10 modulates gene transcription by steroid hormones by promoting the interaction between heat-shock proteins and the ecdysone receptor EcRB1 [51].

Finally, Cdk20 (also known as cell cycle-related kinase (CCRK)) can interact with cyclin H and originally was proposed to have CAK activity for Cdk2, suggesting a close relationship with Cdk7. However, its role as a CAK is controversial [52], and additional data suggest that it functions as an activating kinase for MAK-related kinase/intestinal cell kinase (ICK) [53]. Expression of Cdk20 activates β-catenin-TCF signaling to stimulate cell-cycle progression [54], whereas its inhibition results in accumulation of ICK at the ciliary tips and prevents cell-cycle entry [55] (Figure 4).

## Frontiers

It is abundantly clear that the CDK family is central to multiple signaling pathways controlling transcription and cell-cycle progression. CDKs probably originated as a system to modulate cell-cycle-promoting activity in response to various cellular scenarios. Over the course of evolution, both CDK and cyclin gene families have independently undergone a significant number of functional specializations [7]. Many of the interactions between specific mammalian CDKs and cyclins have been reported *in vitro*. However, the biochemical promiscuity in CDK-cyclin interactions makes it difficult to evaluate properly the *in vivo* physiological relevance of specific CDK-cyclin complexes. For instance, Cdk1 is thought to be activated mainly by A- and B-type cyclins but can also bind to, and be activated by, D- or E-type cyclins in the absence of Cdk4/Cdk6 or Cdk2, respectively [9,27,56]. Cdk5 can also bind to D-type cyclins, although to what extent these complexes are active or relevant *in vivo* is not clear. The situation is even more complex for the lesser-known family members for which there are no current *in vivo* data [2].

Although the comparison of the yeast CDKs has promoted the convenient division between transcriptional and cell-cycle activities, the multiple interactions between these two activities in higher eukaryotes makes it difficult to maintain this simple classification. First, transcription and cell-cycle progression cannot be opposed as these processes function at different layers in cell biology. Arguably, transcription is a major regulatory pathway required for cell-cycle entry. Major cell-cycle-related kinases such as Cdk4 and Cdk6 mostly function by phosphorylating transcription regulators such as Rb or Smads [3,25], and the archetypal cell-cycle kinase Cdk1 also phosphorylates multiple transcription factors and epigenetic modulators (Figure 4) [5]. By contrast, major ‘transcriptional’ CDKs such as Cdk7 or Cdk11 directly control cell-cycle progression, in some instances independently of transcription. Finally, a single CDK can have separate cell-cycle-related and transcriptional activities. As an example, Cdk6 has recently been characterized as a chromatin factor (Figure 4) that regulates transcription factors involved in angiogenesis or the NF-κB pathway [57,58], a process independent of the classical Cdk4/6-cyclin-D-Rb pathway involved in cell-cycle regulation.

As a consequence of their importance in multiple processes, CDKs are frequently mutated or deregulated in disease. A classic example is the almost universal deregulation of the CDK-cyclin-Rb pathway in cell-cycle entry during malignant transformation [25]. Underlining the significance of CDKs, inhibitors of Cdk4 and Cdk6 received in 2013 the Food and Drug Administration ‘breakthrough therapy’ designation for treatment of patients with breast cancer [59]. Other members of the CDK family can also be considered as interesting targets for therapeutics in cancer or other diseases. Cdk5 displays multiple roles in neurodegenerative diseases [28] and in other tissues with relevance to diabetes, cardiovascular disease or cancer [29]. Cdk8 exhibits copy-number gains in colon cancers, and recently it has been characterized as a coactivator of the beta-catenin pathway in colon cancer cell proliferation [60,61]. Cdk10 is a major determinant of resistance to endocrine therapy for breast cancer [62], and inhibition of Cdk12 confers sensitivity to inhibitors of poly (ADP-ribose) polymerases PARP1 and PARP2 [63]. Cdk14 confers motility advantages and metastatic potential in hepatocellular carcinoma motility and metastasis [64,65]. Finally, as indicated above, cyclin Y kinases regulate the Wnt pathway [31], providing new therapeutic opportunities that are yet to be explored. Hence, it seems very likely that new targets within the CDK family will be explored in the near future for therapy of cancer or other diseases.

## Box 1. The cyclin family

Cyclins are a large family of approximately 30 proteins varying in mass from 35 to 90 kDa. These proteins are structurally defined by the presence of the so-called cyclin box, a domain of approximately 100 amino acid residues that forms a stack of five α-helices. Many cyclins have two cyclin boxes, one amino-terminal box for binding to CDKs, and a carboxy-terminal box that is usually required for the proper folding of the cyclin molecule. The cyclin box is also present in other molecules such as the retinoblastoma protein (Rb), the transcription factor TFIIB and Cables (CDK5 and ABL1 enzyme substrate 1), which are unlikely to function as CDK activators. In general, cyclins show less sequence similarity than the CDKs. The cyclin family contains approximately 29 protein in humans, clustered in 16 subfamilies and three major groups: group I (cyclin B group: A-, B-, D-, E-, F-, G, J, I and O); group II (cyclin Y group - a partner of the Cdk5 subfamily); and group III (cyclin C group: C-, H-, K-, L- and T- - major partners of transcriptional CDKs) [7,66]. Cyclin D and cyclin E clades (partners of Cdk1 and Cdk4 subfamilies) have undergone lineage-specific expansion and specialization in metazoa and plants [7].

## Box 2. The CDK-activating kinase complex

The CAK complex (comprising Cdk7, cyclin H and Mat1) phosphorylates the T-loop of all CDKs tested, thus participating in their activation. Furthermore, this complex can be part of the transcription factor phosphorylating the CTD of RNAPII as well as multiple nuclear receptors such as retinoic acid or thyroid receptors, the estrogen receptor α or the vitamin D receptor co-activator Ets1 [33]. The CAK complex can also be found associated with an additional subunit of TFIIH - the DNA-dependent helicase Xpd - forming a complex known as CAK-XPD. This complex plays a role in the coordination and progression of mitosis, likely as a consequence of the redistribution of CAK within different cell compartments during the late nuclear-division steps [67].

## Abbreviations

CAK: CDK-activating kinase; CCRK: Cycle-related kinase; CDK: Cyclin-dependent kinase; CTD: C-terminal domain; DYRK: Dual-specificity tyrosine-regulated kinase; ICK: Intestinal cell kinase; MAPK: Mitogen-activated protein kinase; NSF: N-ethylmaleimide-sensitive factor; PARP: Poly (ADP-ribose) polymerase; P-TEFb: Positive transcription elongation factor b; Rb: Retinoblastoma protein; RNAPII: RNA polymerase II.

## Competing interests

The author declares that he has no competing interests.
